# Case management in primary palliative care is associated more strongly with organisational than with patient characteristics: results from a cross-sectional prospective study

**DOI:** 10.1186/s12904-015-0029-8

**Published:** 2015-07-02

**Authors:** Annicka GM van der Plas, Anneke L Francke, Kris C Vissers, Wim JJ Jansen, Luc Deliens, Bregje D Onwuteaka-Philipsen

**Affiliations:** Department of Public and Occupational Health, VU University Medical Center, P.O. Box 7057, 1007 MB Amsterdam, The Netherlands; Center of Expertise in Palliative Care, VU University Medical Center, P.O. Box 7057, 1007 MB Amsterdam, The Netherlands; NIVEL, Netherlands Institute for Health Services Research, PO Box 1568, 3500 BN Utrecht, The Netherlands; Department of Anaesthesiology, Pain, and Palliative Medicine, Radboud University Nijmegen Medical Centre, PO Box 9101, 6500 HB Nijmegen, The Netherlands; Department of Anaesthesiology, VU University Medical Center, P.O. Box 7057, 1007 MB Amsterdam, The Netherlands; Vrije Universiteit Brussel and Ghent University, End-of-Life Care Research Group, Laarbeeklaan 103, B-1090 Brussels, Belgium

**Keywords:** Case management, End-of-life care, Palliative care, Primary care, Nursing

## Abstract

**Background:**

Case managers have been introduced in Dutch primary palliative care; these are nurses with expertise in palliative care who offer support to patients and informal carers in addition to the care provided by the general practitioner and home care nurses. This study aims to describe support and investigate what characteristics of patients and the organizational setting are related to the number of contacts and to the number of times topics are discussed between the case manager and patients and/or informal carers.

**Methods:**

Prospective study following cancer patients (n = 662) receiving support from a palliative care case manager in Dutch primary care, using registration forms filled out by the case manager after contact with the patient and/or informal carer. In backward linear regression, the association was studied between patient or organizational characteristics and the number of contacts and the number of times conversation topics were discussed.

**Results:**

Organizational characteristics add more to explained variability in data than patient characteristics. Case managers provide support in a flexible manner with regard to the number, mode, persons present, and duration of contacts. Support covered all domains of palliative care, with most attention given to physical complaints, life expectancy and psychological aspects.

**Conclusions:**

Support offered by the case managers is prompted by characteristics of the organization for which they work. This is contradictory to the idea of patient centered care highly valued in palliative care.

## Background

Most people prefer to die at home [1], so the availability of community based palliative care is important to help meet patients’ needs. In the Netherlands, palliative care for home-dwelling patients is mainly provided by generalist care providers i.e. general practitioners (GPs) and home care professionals [2]. The World Health Organization (WHO) [3] stresses that physical, emotional, and spiritual care needs of the patient are all considered important concerns in palliative care. Patients have a broad range of symptoms and it is hard to keep up to date with the new, advanced and complex treatment options now available in palliative care [4–6]. Additionally, GPs and home care nurses may have difficulties or discomfort assessing and discussing prognosis, psychological and spiritual/existential issues [7–9]. Case managers with specific expertise regarding palliative care have been introduced to help patients with palliative care needs and their informal carers obtain the palliative care that matches their preferences [10].

Case managers’ work covers two complementary levels [11]. At an individual level the case manager provides advice or referral to patients and their informal carers. At the level of the organization of care, the case manager has a central position and collaborates with multiple healthcare providers, and provides continuity between professionals and organizations. Tasks can include assessment, planning, implementing, coordinating, monitoring and evaluating the options and services required to meet the client’s health and service needs [12]. The case manager provides support in addition to the care provided by the home care nurse and general practitioner. The organizational affiliation of the case managers in the Netherlands varies; case managers can be employed by a home care organization, by a hospice or by a collaborative venture between institutions (e.g. a home care organization working together with a hospital). Another distinctive feature was their target group; varying from patients from diagnosis onwards to patients in the final stage of their life.

The relationship to the patient should be central in palliative care and support from the case manager should be tailored to the individual needs of the patient [13]. Topics discussed between the case manager and the patient and informal carer should cover physical, emotional, and spiritual care needs of the patient. Important aims of case management are that support is flexible, delivered according to the needs of the patient and informal carer at that moment, and delivered as long as necessary. This should be reflected in characteristics of contacts such as the number of contacts, modes of contact, an duration of contacts. There is paucity in research describing these characteristics for case management in primary palliative care.

Since both palliative care in general [3] and case management in palliative care in particular [13] aim to be highly patient centered, characteristics of patients should be more guiding in content of care than characteristics of the organization providing care. Therefore this study aims to answer the following two questions:

1. What support is provided by the palliative care case manager with regard to number of contacts, mode of contact, duration of contacts, time between the first and last recorded contact, persons present during contacts, and content of contacts?
2. What characteristics of patients and the organizational setting are related to the number of contacts with the patient and to the number of conversations the palliative care case manager has per topic with patients and/or informal carers?

## Methods

### Design

Prospective study in a group of Dutch cancer patients (n = 662) receiving support from a palliative care case manager.

### Setting

The population of the Netherlands is 16.9 million [14]. Each year, about 77,000 people die of non-acute illnesses, 31 % of them dying at home [15]. The number of not-unexpected deaths per GP per year is estimated to be 12–13 on average [16]. Home care nurses who are confronted with end-of-life care see on average 10 palliative care patients a year [17]. There is a wide range of short courses available on palliative care for GPs and home care nurses. Specialized palliative care knowledge is available to GPs and home care nurses through consultation teams operating all over the Netherlands, mainly offering advice by telephone. These teams are consulted approximately 6000 times a year (6 % of the number of not-unexpected deaths) [16]. And in some regions, nurse case managers with specific expertise regarding palliative care have been introduced to visit the patients at home (for a map of the Netherlands with the locations see [10]).

Case management initiatives in primary care were identified in a nationwide survey [10]. Of the 20 initiatives identified in that survey, 13 were investigated in the current study. The term ‘initiative’ is used to do justice to organizational differences, since not all case managers work in a team of case managers; there was one initiative with one case manager, for example, while another case manager is part of a team in which not all members offer case management. See Table 1 for more information on the participating initiatives, and for a more in depth discussion of initiatives please refer to [10]. For the present analysis, we used data about cancer patients (96 % of all referred patients) with at least one registered contact with the case manager (94 % of all referred patients), for whom data collection had stopped before the end of the research period (91 % of all referred patients).

**Table 1:** Characteristics of participating case management initiatives

	Number of initiatives (n = 12)^†^	Percentage of patients (n =662)
Organization offering case management		
- home care organiz (Table uses UK-ENG, text is in UK-ENG)ation	5	48.6 %
- collaboration between institutions^††^	5	28.4 %
- hospice	2	23.0 %
Target group of the initiative		
- from curative care onwards	3	27.8 %
- from life prolonging care onwards	3	23.6 %
- only palliative care patients	6	48.6 %
Number of years the initiative was active/operational at start of the study		
- less than a year	3	11.9 %
- one – five years	7	61.0 %
- five years or longer	2	27.0 %
Number of case managers employed	mean = 3.6 (SD 2.2)	
- one case manager	1	1.7 %
- two case managers	4	50.9 %
- three or four case managers	4	34.9 %
- five or more case managers	3	12.5 %
Number of full time equivalents (fte)	mean = 1.3 (SD 0.8)	
- unknown	1	0.6 %
- 0,5 fte or less	1	1.7 %
- between 0,5 and 1 fte	5	45.7 %
- between 1 and 2 fte	2	24.5 %
- 2 fte or more	3	27.6 %
Number of patients enrolled in the study		
- less than 50 patients	6	6.9 %
- 50 – 100 patients	2	19.9 %
- 100 or more patients	4	73.1 %

^†^Of the 13 participating initiatives, one was specifically focussed on patients with COPD and was not included in this paper (only initiatives involving cancer patients were included in this paper)

^††^An example of a collaboration of institutions is a hospital working together with a home care organisation

**Table 2:** Characteristics of patients receiving support from a case manager

	Total (n = 662)^†^
	n (%)
Sex, male	328 (49.5)
Age, mean (SD)	66.8 (12.3)
Type of cancer	
- lung	174 (26.6)
- colon	85 (13.0)
- breast	75 (11.5)
- other	319 (48.9)
At least one additional diagnosis	269 (42.4)
Treatment aims	
- mainly palliative treatment aims	170 (26.6)
- mainly curative or life prolonging treatment aims	25 (3.9)
- combined treatment aims	445 (69.5)
Functional Status (ECOG)	
- fully functional	52 (8.0)
- limited to small/light activities	230 (35.4)
- bedridden less than 50 %	140 (21.5)
- bedridden more than 50 %	149 (22.9)
- fully in need of support	79 (12.2)
Life expectancy of patient at start of case management, estimation given	345 (52.2)
Life expectancy of patient when estimated	
- less than 3 months	92 (26.7)
- 3 to 6 months	100 (29.0)
- 6 months or longer	153 (44.3)
Residential circumstances	
- with partner and children	90 (13.8)
- with partner	350 (53.7)
- with children	25 (3.8)
- alone	161 (24.7)
- other (e.g. living in an institution or temporarily living with family)	26 (4.0)
Informal carers (more than 1 answer possible)	
- none	7 (1.1)
- partner	401 (61.8)
- children	444 (68.5)
- other family	200 (30.9)
- other (e.g. friends, neighbours)	225 (34.7)

^†^ Number of missing observations between 0 and 27

**Table 3:** Characteristics of contacts of the case manager with patients and/or carers

	Total (n = 662)
	n (%)
Number of contacts with patient and/or informal carer, median (range)	4.0 (1–36)
- one	104 (15.7)
- two – five	297 (44.9)
- six - ten	160 (24.2)
- eleven - twenty	85 (12.8)
- twenty-one or more	16 (2.4)
Number of contacts with patient only, median (range)	0 (0–30)
Number of contacts with informal carer only, median (range)	1 (0–19)
Number of contacts with both patient and informal carer, median (range)	2 (0–18)
Mode of contact	
- visits, median (range)	2.0 (0–23)
- by telephone, median (range)	2.0 (0–19)
Duration of contact in minutes by mode	
- visits, median (range)	60.0 (2–190)
- by telephone, median (range)	15.0 (2–120)
Time between first and last recorded contact in days	
- zero	108 (16.3)
- up to one week	38 (5.7)
- up to one month	128 (19.3)
- one to three months	143 (21.6)
- three to six months	131 (19.8)
- half year to a year	84 (12.7)
- more than a year	30 (4.5)

**Table 4:** Content of contacts of the case manager with patients and/or carers

	Number of times the patient/carer had…	Number of patients/carers who had at least once…
	(n = 662)	(n = 662)
	median (range)	n (%)
A conversation about:		
- physical complaints	3.0 (0–26)	619 (93.5)
- life expectancy	1.0 (0–16)	526 (79.5)
- psychological aspects	2.0 (0–22)	525 (79.3)
- incurability of disease	1.0 (0–13)	502 (75.8)
- medical treatment(s)	1.0 (0–17)	456 (68.9)
- possibilities of palliative care	1.0 (0–12)	456 (68.9)
- social aspects	1.0 (0–22)	443 (66.9)
- main diagnosis	1.0 (0–11)	432 (65.3)
- burden of treatment(s)	1.0 (0–15)	413 (62.4)
- spiritual aspects	0 (0–12)	312 (47.1)
- possible medical complications	0 (0–7)	274 (41.4)
- other	0 (0–12)	302 (45.6)
Been given information on:		
- care services	1.0 (0–13)	468 (70.7)
- illness	1.0 (0–13)	441 (66.6)
- nursing / physical care	1.0 (0–16)	403 (60.9)
- medical treatment(s)	1.0 (0–15)	354 (53.5)
- coping	1.0 (0–14)	347 (52.4)
- home care technology	0 (0–6)	215 (32.5)
- other	0 (0–13)	296 (44.7)
An assessment of care needs	2.0 (0–22)	577 (87.2)
Coordination of care	0 (0–15)	318 (48.0)

### Questionnaires

A questionnaire was filled out by the case manager at the moment of referral of a patient to the case manager. It contained structured questions regarding characteristics of the patient, such as demographic data and questions on diagnosis and prognosis.

A registration form was filled out by the case manager after each contact with the patient and/or informal carer. It contained structured questions on the contact such as mode (phone, visit or other) location and attendees, content of the contact (dichotomous questions on topics of conversation and actions of the case manager during the contact), and the duration of the contact.

Both questionnaires were drafted to study implementation and support provided by the case manager. The questionnaires were piloted on a small sample of respondents to ascertain that questions were clearly formulated and relevant.

### Ethical considerations

Under Dutch law this study is exempt from approval from an ethics committee since it did not involve imposing any interventions or actions [18]. Data were anonymized by the case manager before being handed over to the authors. The authors provided information material about the study to the case managers, so they could inform the patients on the study and get consent.

### Procedure

Data were gathered from March 2011 until the end of 2013. When a patient was referred to the case manager, the case manager filled out a questionnaire. For every contact the case manager had with the patient and/or informal carer, a registration form was filled out. The questionnaire and registration forms used the same unique patient identification number. When a period of ‘silence’ (not returning registration forms) ensued after a patient was entered into the study, the researcher asked the case manager whether provision of support was still ongoing. Initiatives with many patients could include every second person in the study instead of every patient, for time management reasons. When not including all patients, case managers were stressed not to ‘choose’ the patients they included in the study, but to keep strictly to the ‘every second patient rule’.

### Data analysis

Frequencies were calculated to describe patients, characteristics of contacts, and content of contacts. To explore if there were any patient or organizational characteristics associated with the number of contacts and the number of times conversation topics were discussed during contacts, backward linear regression (removal at p < .05) was performed. The number of contacts and the number of conversations per topic were skewed, but did not follow a Poisson distribution (variance was larger than the mean in all variables). Therefore the log transformation of the number of contacts and the number of conversations per topic were used in the model [19]. Logtransformed data are to be interpreted like odds ratios (even when they are transformed back like in Table 5). To reduce the number of missing observations after logtransformation, a fixed number (1) was added to the number of contacts and the number of conversations per topic before logtransformation [19]. Separate regression models were fitted for each conversation topic. The following patient characteristics were entered into the analysis: age, sex, living situation of the patient (alone or not), whether the patient had an additional diagnosis (none versus at least one), functional status, and starting point of case management (early or later in disease trajectory). The following organizational characteristics were entered into the analysis: organization where the case manager was employed (home care organization, hospice, or collaboration between institutions), target group of the initiative (patients receiving support from the case manager from diagnosis onwards, patients receiving life prolonging or palliative care, patients receiving palliative care). To investigate the separate contribution of patient characteristics and organizational characteristics, these were added as separate blocks in the regression models. To control for the number of contacts, this variable was added as a first block to the models investigating the conversation topics. As a measure of the goodness of fit of the models, the value of R^2^ is used to determine the proportion of variability in a data set that is accounted for by the statistical model (reported in Table  6). Data were analyzed using SPSS, IBM Statistics for Windows version 20.0.

**Table 5:** Statistically significant relationships between number and content of contacts and patient and organizational characteristics^†^

	Patient						Organization				Care
							Affiliation of the case manager:		Target group:		
	Age^††^	Sex female^††^	Living situation alone^††^	At least one additional diagnosis^††^	Functional status^††^	Start of case manage-ment^††^	Home care organi-zation^††^	Hospice^††^	From life prolonging care onwards^††^	Only palliative care patients^††^	Number of contacts
Number of contacts		1.12 (1.01 – 1.24)			0.89 (0.85 – 0.93)	1.22 (1.08 – 1.38)	0.54 (0.41 – 0.71)	0.57 (0.50 – 0.66)	0.71 (0.62 – 0.82)	0.65 (0.49 – 0.86)	
Number of conversations about:											
- physical complaints					0.96 (0.93 – 0.98)		1.47 (1.37 – 1.58)	1.52 (1.40 – 1.65)			1.12 (1.12 – 1.13)
- psychological aspects			0.87 (0.79 – 0.96)		0.95 (0.91 – 0.98)		1.22 (0.97 – 1.53)	1.14 (1.01 – 1.28)	0.87 (0.77 – 0.98)	1.01 (0.80 – 1.27)	1.10 (1.09 – 1.11)
- life expectancy									1.40 (1.25 – 1.56)	1.04 (0.95 – 1.14)	1.08 (1.07 – 1.09)
- incurability of disease							1.69 (1.37 – 2.10)	0.90 (0.81 – 1.01)	1.54 (1.38 – 1.73)	1.68 (1.34 – 2.09)	1.08 (1.07 – 1.08)
- possibilities of palliative care	1.01 (1.00 – 1.01)		0.90 (0.82 – 0.99)			0.81 (0.73 – 0.89)	1.08 (0.98 – 1.20)	1.47 (1.31 – 1.67)			1.07 (1.06 – 1.07)
- medical treatment(s)	1.00 (0.99 – 1.00)				0.92 (0.89 – 0.96)	1.24 (1.12 – 1.37)	1.48 (1.19 – 1.86)	1.20 (1.07 – 1.36)	1.16 (1.03 – 1.30)	1.13 (0.90 – 1.42)	1.08 (1.07 – 1.09)
- social aspects					0.94 (0.91 – 0.98)	0.89 (0.79 – 1.00)	1.14 (0.88 – 1.48)	1.39 (1.21 – 1.59)	0.74 (0.65 – 0.85)	1.07 (0.82 – 1.40)	1.07 (1.06 – 1.08)
- main diagnosis						1.09 (1.00 – 1.18)	1.84 (1.52 – 2.23)	0.95 (0.86 – 1.06)	1.36 (1.23 – 1.50)	1.22 (1.00 – 1.49)	1.03 (1.03 – 1.04)
- burden of treatment(s)	0.99 (0.99 – 1.00)		1.11 (1.00 – 1.23)		0.93 (0.89 – 0.97)	1.22 (1.09 – 1.36)	1.62 (1.27 – 2.06)	1.52 (1.33 – 1.73)	1.25 (1.10 – 1.43)	1.21 (0.94 – 1.56)	1.07 (1.06 – 1.08)
- spiritual aspects			0.88 (0.80 – 0.97)						0.79 (0.70 – 0.86)	0.90 (0.82 – 0.99)	1.05 (1.04 – 1.06)
- possible medical complications							1.35 (1.24 – 1.47)	1.27 (1.15 – 1.40)			1.04 (1.04 – 1.05)

^†^ N = 662, number of missing values range from 0 to 24. Reported are unstandardized regression coefficients with 95 % confidence intervals. All dependent variables were logtransformed due to skewed data, and can therefore be interpreted like odds ratios. In the presented models all variables have p-values of 0.05 or below (the affiliation of the case manager and the target group of the organization are both nominal variables, all categories are reported when at least one of them has a p-value of 0.05 or below). ^††^ Reference groups in analyses: Sex male = reference; Living situation not alone = reference; No additional diagnosis = reference; Start of case management late in disease trajectory = reference; Affiliation is collaboration between institutions = reference; Target group of organization from curative care onwards = reference. For functional status higher score = lower status (in Table 4; the higher the score and therefore the lower the status, the less conversations on a topic)

**Table 6:** Proportion of variability that is accounted for by the statistical model

	Number of contacts (Block 1)	Block 1 and patient characteristics (Block 2)	Final model^†^: Block 2 and organization characteristics (Block 3)
	R^2^	R^2^	R^2^
Number of contacts	NA	0.063	0.170
Number of conversations about:			
- physical complaints	0.661	0.670	0.727
- psychological aspects	0.463	0.478	0.491
- life expectancy	0.345	NA	0.386
- incurability of disease	0.316	NA	0.410
- possibilities of palliative care	0.239	0.265	0.317
- medical treatment(s)	0.354	0.431	0.472
- social aspects	0.277	0.289	0.358
- main diagnosis	0.065	0.080	0.365
- burden of treatment(s)	0.239	0.327	0.389
- spiritual aspects	0.190	0.198	0.217
- possible medical complications	0.145	NA	0.205

^†^ This is the model presented in Table 5

## Results

### General characteristics of patients

Patients had a mean age of 66.8 years (SD 12.3; range 29–98), and half of patients were male (49.5 %) (Table 2). A quarter (26.6 %) had a diagnosis of lung cancer, and 42.4 % of patients had at least one other diagnosis besides cancer. Most (69.5 %) patients had a combination of treatment aims when they entered case management, 3.9 % had cure or life prolongation as treatment aim and 26.6 % had a palliative care treatment aim. Also, 8.0 % of patients were fully functional at start of case management, 35.4 % was limited to light activities, and 21.5 % of patients was bedridden for less than half a day. For half (52.2 %) of the patients, an estimation of life expectancy was given at start of support by the case manager, and when given, 44.3 % of patients had an estimated life expectancy of six months or longer. Patients were mostly living with a partner (53.7 %) or alone (24.7 %), a further 13.8 % lived with partner and children. Most mentioned informal carers were the partner (61.8 %) and children (68.5 %) of patients.

### Characteristics of the contacts between the case manager and patient and/or informal carer

The number of contacts ranged from 1 to 36 (Table 3), with a median of 4 contacts. Contacts were mostly with the patient and informal carer together. Both home visits and telephone contacts occurred, with telephone calls being shorter in duration than home visits.

### Content of contacts

The topics discussed at least once with most patients and/or informal carers were physical complaints (93.5 % of patients/informal carers), life expectancy (79.5 % of patients/informal carers), and psychological aspects of being ill (79.3 % of patients/informal carers) (Table 4). The information given at least once to most patients and/or informal carers was on care services, illness and nursing or physical care. Physical complaints and psychological aspects were discussed most often per patient.

### Patient and organizational characteristics associated with number and content of contacts

The number of contacts was higher for female patients; female patients and/or their informal carers had 12 % (B = 1.12, CI 1.01–1.24) more contacts with a case manager than male patients (Table 5). Lower functional status (B = 0.89, CI 0.85–0.93) was associated with fewer contacts and first contact early in the disease trajectory (B = 1.22, CI 1.08–1.38) was associated with more contacts. Case managers from a home care organization (B = 0.54, CI 0.41–0.71) or from a hospice (B = 0.57, CI 0.50–0.66) had fewer contacts with patients compared to case managers from a collaboration between institutions. Organizations with a target group of patients receiving either life prolonging care and/or palliative care (B = 0.71, CI 0.62–0.82) and organizations targeting palliative care patients only (B = 0.65, CI 0.49–0.86) had fewer contacts with patients compared to organizations whith a target group of patients receiving care from diagnosis onwards. The relation between discussion topics, patient characteristics, and organizational characteristics is detailed in Table 5.

### Contribution of patient and organizational characteristics to the models

Models on conversation topics which included number of contacts and organizational characteristics consistently explained most variability in data (Table 6). Patient characteristics did not contribute to the explanation of variability in data in conversations on life expectancy, incurability of disease and possible medical complications. Furthermore, in other conversation topics, patient characteristics contributed relatively little to explanation of variability in data, only in three models did they add more to explain variability than organizational characteristics.

## Discussion

Organizational characteristics are important in prediction of the number of times topics are discussed with patients; they add more to explained variability in data than patient characteristics. Differences were most articulate between organizations targeting patients from diagnosis onwards and organizations targeting patients receiving life prolonging and/or palliative care. Furthermore, case managers working from a hospice and from a home care organization have more conversations on topics than case managers from a collaboration between institutions. Case managers provide support in a flexible manner with regard to the number, mode, persons present, and duration of contacts. Time between the first and last contact also varied. Support covered all domains of palliative care, with most attention given to physical complaints, life expectancy and psychological aspects of being ill.

### Number and content of contacts

The variability in number of contacts and content of contacts could be an indication that support is offered according to the patient’s needs or wishes. However, we did not study who initiated or requested actions and conversations during contacts. Assessment of specialist palliative care nurses with regard to quality of life may differ from assessment by patients [20], and also perceived needs of the patient and informal carer may differ between nurses and patients [21]. So, it could be that different topics would ensue when the case managers initiated the actions as compared to when patients and informal carers initiated actions. However, it is likely that both the case manager and the patient and/or informal carer had an influence on the kind of support given. In a study on patients’ view on the specialist palliative care nurse [22], patients valued the nurses’ work, particularly their advice on practical matters, information given about their disease, emotional support, advice on symptoms, and help with communication. The persons who refer patients to palliative care case managers expect psychosocial support to be given, since this was mentioned as a reason for referral in more than three quarters of patients with a combination of treatment aims and in two third of patients with a sole palliative care treatment aim [23].

Functional status of the patient at the start of support by the case manager and start of case management early or late in disease trajectory are the patient characteristics most often related to conversation topics. Since models are controlled for the number of contacts, this is not simply an issue of opportunity (more chance of discussing topics when there is more time). Patients for whom the start of case management was early in the disease trajectory more often had conversations on medical treatments, main diagnosis and burden of treatment; all three topics may be particularly relevant to patients still receiving life prolonging or curative treatment. Likewise, possibilities of palliative care may have been discussed less with patients for whom support by the case manager started early, because they were still focused on life prolonging or curative treatment. In a study on palliative care by the GP, having end-of-life conversations was related to the provision of palliative care and not with functional status [7].

### Importance of organizational characteristics in the provision of support by the case manager

With regard to target group of the organization, the biggest differences were found between case managers working from an organization targeting patients from diagnosis (curative care) onwards compared to case managers working from an organization targeting patients receiving life prolonging and/or palliative care. It is remarkable that the difference is not bigger between organizations targeting patients receiving palliative care only compared to organizations targeting patients from diagnosis (curative care) onwards. Specific attention is paid to patients receiving life prolonging care, not just in line with a continuously heightened or intensified attention to discussion topics during the process of treatment from curative to ultimately terminal care. More attention is paid to life expectancy, incurability of disease, medical treatments, main diagnosis and burden of treatment, but conversations on psychological, social and spiritual aspects occur less with case managers from an organization targeting patients receiving life prolonging and/or palliative care (compared to organizations targeting patients from diagnosis onwards).

The number of conversations per topic was higher for case managers working from a home care organization and a hospice, compared to case managers from a collaboration between institutions. Again, since models are controlled for the number of contacts, this is not simply an issue of opportunity. Differences in palliative care between settings may be explained by differences in availability of care and culture [24, 25], but current findings are all within the primary care setting. Organizational aspects played a role in provision of advance care planning in community-based care management organizations [26]; in that study, amongst other things availability of training and resources was linked to advance care planning. It is notable that characteristics of the organization for which the case manager works add more to explain the number of conversation per topic than patient characteristics. Further research is needed to determine why some case managers within the primary care setting discuss topics less or more often, depending on the organization they work for. Aspects that should be taken into account in future research may be: whether some case managers go less in depth and are therefore able to discuss topics more often, whether they are more efficient with their time, and how much time during contacts is spent on conversations, providing information and care coordination.

### Strengths and limitations of this study

This study is an important step in opening the ‘black box’ of case management in palliative care [27]. This is the first study to investigate the relationship between organizational characteristics of primary palliative care case management and provision of support. Information on contacts was gathered continuously during support from the case manager; recall bias therefore will be low. However, our study has some potential limitations that should be kept in mind. This study is conducted within the Dutch health care system where primary palliative care is mostly delivered by generalist care providers (the GP and home care nurses), with case managers offering additional support. This could influence the way case management is delivered and the topics discussed. The patient characteristics used in our analyses were found relevant for prediction of service use in previous studies [28–30]. However, they may not be suitable specifically for patients receiving case management. Future studies should explore whether other patient characteristics than those used would better predict the number of contacts and conversation topics; for instance characteristics better detailing the complexity of the home or medical situation of the patient may be more appropriate.

## Conclusion

Case managers provide support in a flexible manner and support covered all domains of palliative care. Despite the generally agreed upon goal of palliative care providing patient centered care, our data suggest that characteristics of the organization are more important in prediction of what topics are discussed between the case manager and the patients and informal carers than patient characteristics. So even though case managers provide support in a flexible manner, this flexibility is ‘colored’ by organizational characteristics. It is notable that organizational characteristics are guiding in care provision, but it is impossible to make recommendations without further research.

## Abbreviations

GP: general practitioner
WHO: World Health Organization
